# The association of off-hour vs. on-hour intensive care unit admission time with mortality in patients with cardiogenic shock: a retrospective multi-centre analysis

**DOI:** 10.1093/ehjacc/zuae012

**Published:** 2024-02-02

**Authors:** Dominik Naumann, Julius Fischer, Jonas Gmeiner, Enzo Lüsebrink, Benedikt N Beer, Maximilian Grieger, Atakan Giousouf, Benedikt Schrage, Christopher Stremmel, Steffen Massberg, Martin Orban, Clemens Scherer

**Affiliations:** Department of Medicine I, LMU University Hospital, LMU Munich, Marchioninistraße 15, 81377 Munich, Germany; DZHK (German Centre for Cardiovascular Research), Munich Heart Alliance, Munich, Germany; Department of Medicine I, LMU University Hospital, LMU Munich, Marchioninistraße 15, 81377 Munich, Germany; Department of Medicine I, LMU University Hospital, LMU Munich, Marchioninistraße 15, 81377 Munich, Germany; Department of Medicine I, LMU University Hospital, LMU Munich, Marchioninistraße 15, 81377 Munich, Germany; Department of Cardiology, University Medical Center Hamburg-Eppendorf, Martinistraße 52, 20251 Hamburg, Germany; DZHK (German Centre for Cardiovascular Research), Hamburg/Lübeck/Kiel, Germany; Department of Medicine I, LMU University Hospital, LMU Munich, Marchioninistraße 15, 81377 Munich, Germany; Department of Medicine I, LMU University Hospital, LMU Munich, Marchioninistraße 15, 81377 Munich, Germany; Department of Cardiology, University Medical Center Hamburg-Eppendorf, Martinistraße 52, 20251 Hamburg, Germany; DZHK (German Centre for Cardiovascular Research), Hamburg/Lübeck/Kiel, Germany; Department of Medicine I, LMU University Hospital, LMU Munich, Marchioninistraße 15, 81377 Munich, Germany; Department of Medicine I, LMU University Hospital, LMU Munich, Marchioninistraße 15, 81377 Munich, Germany; DZHK (German Centre for Cardiovascular Research), Munich Heart Alliance, Munich, Germany; Department of Medicine I, LMU University Hospital, LMU Munich, Marchioninistraße 15, 81377 Munich, Germany; Department of Medicine I, LMU University Hospital, LMU Munich, Marchioninistraße 15, 81377 Munich, Germany; DZHK (German Centre for Cardiovascular Research), Munich Heart Alliance, Munich, Germany

**Keywords:** Cardiogenic shock, VA-ECMO, Nightshift, Off-hour, Clinical-trial

## Abstract

**Aims:**

Studies have shown a so-called off-hour effect for many different diseases, but data are scarce concerning cardiogenic shock. We therefore assessed the association of off-hour vs. on-hour intensive care unit admission with 30-day mortality in patients with cardiogenic shock.

**Methods and results:**

In total, 1720 cardiogenic shock patients (666 admitted during off-hours) from two large university hospitals in Germany were included in retrospect. An admission during off-hours was associated with increased 30-day mortality compared to an admission during on-hours [crude mortality 48% vs. 41%, HR 1.17 (1.03–1.33), *P* = 0.017]. This effect remained significant after propensity score matching (*P* = 0.023). Neither patients with a combined SCAI stage D and E (*P* = 0.088) or C (*P* = 0.548) nor those requiring cardiopulmonary resuscitation (*P* = 0.114) had a higher mortality at off-hour admission. In contrast, those without veno-arterial extracorporeal membrane oxygenation [HR 1.17 (1.00–1.36), *P* = 0.049], without acute myocardial infarction [HR 1.27 (1.02–1.56), *P* = 0.029] or a with combined SCAI stage A and B [HR 2.23 (1.08–4.57), *P* = 0.025] had an increased mortality at off-hour admission.

**Conclusion:**

Our study showed an increased mortality in patients with cardiogenic shock admitted during off-hours, especially in those with a milder onset of disease. This stresses the importance of a thorough workup of each patient, especially at times of limited resources, the menace of underestimating the severity of cardiogenic shock, and the need for an improved 24×7 available risk stratification.

## Graphical Abstract

**Graphical Abstract:**
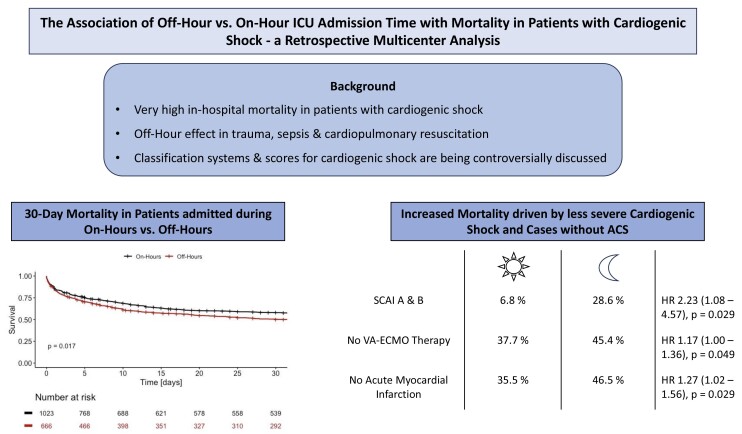

## Introduction

Cardiogenic shock is a syndrome describing the most severe form of acute heart failure defined by clinical and biochemical signs of hypoperfusion with an in-hospital mortality of up to 50%.^1,2^ In prior studies the off-hour effect on mortality has been discussed controversially for different diseases, especially in acute coronary syndrome. A meta-analysis by Yu et al. compared 45 articles and found a increased short and long-term mortality in patients with acute myocardial infarction admitted during off-hours.^3^ Another meta-analysis showed that these patients not only had an increased mortality but also an increased door-to-balloon-time resembling a delay in treatment.^4^ However, these findings are controversial as other studies could not reproduce them.^5,6^ Nevertheless, in different diseases such as intracranial bleeding the prior described off-hour effect could also be shown.^7^ Interestingly, one study concerning trauma patients showed an increased mortality during daytime and business days compared to nighttime and non-business days.^7^ Furthermore, Aoki et al. presented a lower adherence to the Surviving Sepsis Campaign bundle in patients with septic shock during the daytime.^8,9^ Therefore the question emerges whether the mortality in cardiogenic shock differs between patients admitted during on-hours and off-hours as data in this field are scarce. One study assessing the mortality in cardiogenic shock depending on the time of admission is a sub-study of the randomized controlled CULPRIT-SHOCK trial showing an equal 30-day and 1-year mortality in patients with myocardial infarction complicated by cardiogenic shock.^10^ Cerbin et al. published a subanalysis of the ASCEND-HF trial, which analyzed the effectiveness of Nesiritide in decompensated heart failure, and found that patients being admitted during off-hours for acute heart failure had a decreased 30 and 180-day mortality compared to those presenting during regular hours.^11^ One has to bear in mind though that these were patients included in prospective studies and therefore a selection and performance bias needs to be taken into consideration concerning both subanalyses. Two other studies were solely looking at subtypes of cardiogenic shock with one analyzing patients with in-hospital cardiac arrest and showing an increased mortality during off-hours.^12^ The second study by Gómez-Sánchez et al. did not find a difference in mortality between on- and off-hour cannulation in cardiogenic shock patients requiring the implantation of a venoarterial extracorporeal membrane oxygenation (VA-ECMO).^13^ We, therefore, aim to answer the question of whether patients with cardiogenic shock irrespective of its aetiology presenting during off-hours have increased 30-day mortality compared to those being admitted during on-hours.

## Methods

This retrospective multi-centre cohort study was conducted in accordance with the declaration of Helsinki and after approval of the local ethic committees (IRB approval number: 18-001).

In order to answer the question of an increased mortality in patients with cardiogenic shock presenting during off-hours, all patients being treated for cardiogenic shock in the cardiac intensive care unit (ICU) wards at LMU University Hospital Munich as well as University Medical Center Hamburg-Eppendorf between 2009 and 2022 were screened.^14^ Both university hospitals offer 24-h availability of cardiac catheterization laboratories as well as implantation of temporary mechanical cardiocirculatory support including VA-ECMO at any time. In both locations, a senior physician with specialized training in intensive care medicine is available on the ward during daytime hours. During off-hours a physician with specialized training in intensive care medicine as well as an interventional cardiologist is on call or at site and at weekends the senior intensivist on call joins the daily ward round. Cardiogenic shock was defined according to the ESC criteria as a systolic blood pressure <90 mmHg for at least 30 min or the need for vasopressors to achieve adequate blood pressure, a cardiac index <1.8 L/min/m^2^, pulmonary congestion or elevated LV filling pressures or the occurrence of signs of impaired organ perfusion (e.g. altered mental status, cold and clammy skin, oliguria and a lactate >2 mmol/L). Exclusion criteria were age under 18 years as well as incomplete data concerning the admission time. On-hours were defined from 08:00 a.m. to 06.59 p.m. and off-hours accordingly from 07.00 p.m. to 07.59 a.m., which resembles day and night shifts at both study sites and also corresponds with times used in prior studies.

All data were extracted from paper-based or electronic case report forms into a web-based registry form (LMUshock registry, World Health Organization trial ID DRKS00015860). Patient data like age, gender, and survival time since admission were collected. In terms of clinical data, the aetiology of cardiogenic shock, SCAI stage, and the need for cardiopulmonary resuscitation (CPR), vasopressors, VA-ECMO, or dialysis were evaluated. The SCAI stage was defined according to consensus statement by Baran et al.^15^ Furthermore, the occurrence of any bleeding Bleeding Academic Research Consortium (BARC) ≥3, stroke or intracranial bleeding, and sepsis during the ICU stay was documented. Laboratory values such as serum lactate, pH, haemoglobin, and creatinine were collected.

The primary outcome was defined as the 30-day mortality stratified by an admission during on-hours or off-hours. Secondary outcomes included the need for extracorporeal life support, the individual SCAI stage, and an acute myocardial infarction.

All statistical analyses were performed using R (Version 4.2.2, Vienna, Austria). Nominal data are presented as absolute values with percentages while continuous data are shown as median with interquartile range. After using a Shapiro–Wilk test to assess the normality, data were analyzed with the Mann–Whitney-U Test. In order to visualize a difference in 30-day mortality, Kaplan–Meier plots were used and compared using the log-rank test. Apart from that, hazard ratios were assessed using Cox regression analyses. Furthermore, we analyzed the number of admission and mortality over the course of a day with an approximation curve and the Mann–Kendall Trend test. To assess the correlation of admission time (on- vs. off-hours) with the 30-day mortality rate, a multivariable Cox regression analysis was used with the covariates of age, sex, and the need for CPR. In order to reduce the chance of possible bias we furthermore performed propensity score matching using the matchit package in R and a logistic regression model with pH, lactate, and CPR as well as age and gender as variables with full matching.^16,17^*P*-Values of <0.05 were considered statistically significant.

## Results

In total 1720 patients were included in the registry according to the prior defined criteria. Of these, 30 had to be excluded due to missing data concerning the exact admission time. As shown in *Table 1*, the median age was 67–68 years and 74% were male. In terms of cardiogenic shock severity, most patients presented with an SCAI stage E (45% at on-hours, 48% at off-hours), followed by C (33 and 30%, respectively) and D (both 16%). There was a significant difference in patients requiring CPR with more resuscitated patients being admitted during off-hours (60% vs. 51%, *P* = 0.001). Almost all patients had a need for vasopressors, additionally a VA-ECMO was implanted in 29% of those admitted during off-hours and 26% of patients presenting during on-hours. One third of the patients in both groups required temporary renal replacement therapy. Concerning the laboratory values, the initial arterial lactate was significantly higher in patients admitted between 7 p.m. and 7.59 a.m. (5.6 vs. 3.7 mmol/L, *P* < 0.001) while creatinine and haemoglobin did not differ between the two groups. In the further course of the ICU stay around 6% developed intracranial bleeding, a stroke was diagnosed in 9% of patients admitted during off-hours and 11% admitted during daytime. Furthermore, a total of 40% in either group had any type of bleeding complicating the clinical course of disease and 14–16% of patients developed a sepsis.

**Table 1:** Patient characteristics

	*Off-*hour *(n = 666)*	*On-*hour *(n = 1024)*	*P-*value
Age (years)	67 (56–77)	68 (56–78)	0.43
Sex			0.71
Male (*n*)	493 (74%)	753 (73.5%)	
Female (*n*)	173 (26%)	270 (26.4%)	
SCAI stage			0.84
NA (*n*)	12 (1.8%)	16 (1.6%)	
A (*n*)	3 (0.5%)	8 (0.8%)	
B (*n*)	25 (3.8%)	37 (3.6%)	
C (*n*)	201 (30.2%)	337 (32.9%)	
D (*n*)	103 (15.5%)	162 (15.8%)	
E (*n*)	322 (48.3%)	464 (45.3%)	
Cardiopulmonary Resuscitation (*n*)	396 (59.5%)	518 (50.6%)	<0.01*
Vasopressors and inotropes (*n*)	608 (91.3%)	941 (91.9%)	0.47
VA-ECMO (*n*)	195 (29.3%)	265 (25.9%)	0.37
Dialysis (*n*)	203 (30.5%)	284 (27.7%)	0.44
Laboratory values			
pH	7.28 (7.19– 7.36)	7.32 (7.21–7.39)	<0.01*
Lactate (mmol/L)	5.6 (2.5–11.1)	3.7 (2.0–9.7)	<0.01*
Creatinine (mg/dL)	1.49 (1.10–2.02)	1.50 (1.10–2.10)	0.99
Haemoglobin (g/dL)	10.93 (9.53–12.82)	10.76 (9.43–12.60)	0.21
Vital parameters			
Heart rate (bpm)	96 (80–113)	98 (82–116)	0.34
Systolic blood pressure (mmHg)	85 (75–99)	85 (75–100)	0.56
Diastolic blood pressure (mmHg)	50 (40–56)	49 (40–55)	0.74
Intracranial bleeding (*n*)	37 (5.6%)	65 (6.3%)	0.51
Ischaemic stroke (*n*)	53 (8%)	97 (9.5%)	0.49
Hemorrhagic stroke (*n*)	3 (0.5%)	13 (1.3%)	0.17
Bleeding BARC *≥*2 (*n*)	270 (40.5%)	401 (39.2%)	0.67
Sepsis (*n*)	108 (16.2%)	146 (14.3%)	0.22

All values are presented as median and confidence intervals or absolute values and percentages. SCAI Classification, Society for Cardiovascular Angiography & Interventions Stages of Cardiogenic Shock; VA-ECMO, venoarterial extracorporeal membrane oxygenation; pH, potential of hydrogen; BARC, Bleeding Academic Research Consortium. *P-values <0.05 were considered statistically significant.

Concerning the aetiology of cardiogenic shock results of further characterization of a subgroup within our cohort with available data are shown in *Table 2*. Most cardiogenic shocks were a result of acute coronary syndrome (58% during off-hour vs. 45.9% on-hour). Underlying arrhythmias were more common in the off-hour cohort (11.3 vs. 9.1%) while cardiomyopathies (18.3 vs. 10.9%) and valvular diseases (11.4 vs. 7.2%) as the cause of cardiogenic shock were seen more frequently with an admission during on-hours.

**Table 2:** Aetiologies of cardiogenic shock

	Off-hour (*n* = 540)	On-hour (*n* = 860)
Arrhythmia	61 (11.3%)	78 (9.1%)
Cardiomyopathy	59 (10.9%)	157 (18.3%)
Pulmonary embolism	9 (1.7%)	21 (2.4%)
Myocarditis	15 (2.8%)	17 (2.0%)
Acute coronary syndrome	313 (58.0%)	395 (45.9%)
Pericardial Tamponade	17 (3.2%)	58 (6.7%)
Valvular	39 (7.2%)	98 (11.4%)
Other	27 (5.0%)	36 (4.2%)

All values are presented as absolute values and percentages.

**Figure 1:**
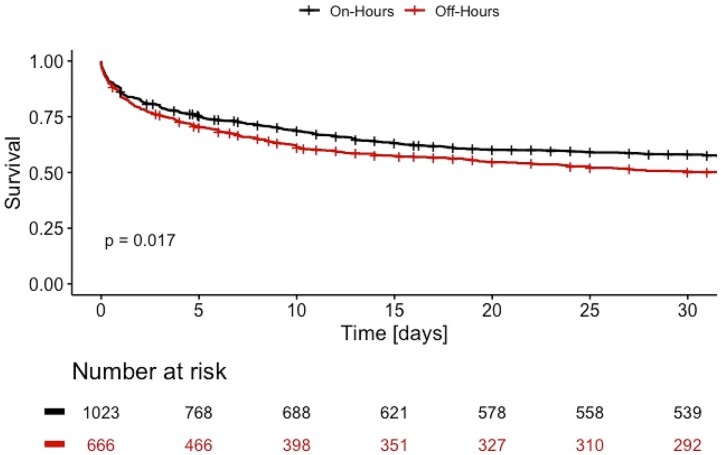
Cumulative survival curves for patients with cardiogenic shock admitted during on-hours (black) and off-hours (red) for 30 days after admission. Each vertical line resembles the maximum survival time in the database for a patient alive.

Addressing the primary endpoint, 423 patients (41%) of those admitted during on-hours had a fatal course of disease after 30 days, and respectively, 322 patients (48%) of those presenting at off-hours. As shown in *Figure 1*, there is a significantly increased 30-day mortality rate in patients with cardiogenic shock being admitted between 7 p.m. and 7.59 a.m. [HR 1.17 (1.03–1.33), *P* = 0.017]. However, there was no discernible difference in mortality between each day of the week for on-hour and off-hour duty in a subgroup of patients, respectively (see Supplementary material online, *Figure S1*).

**Figure 2:**
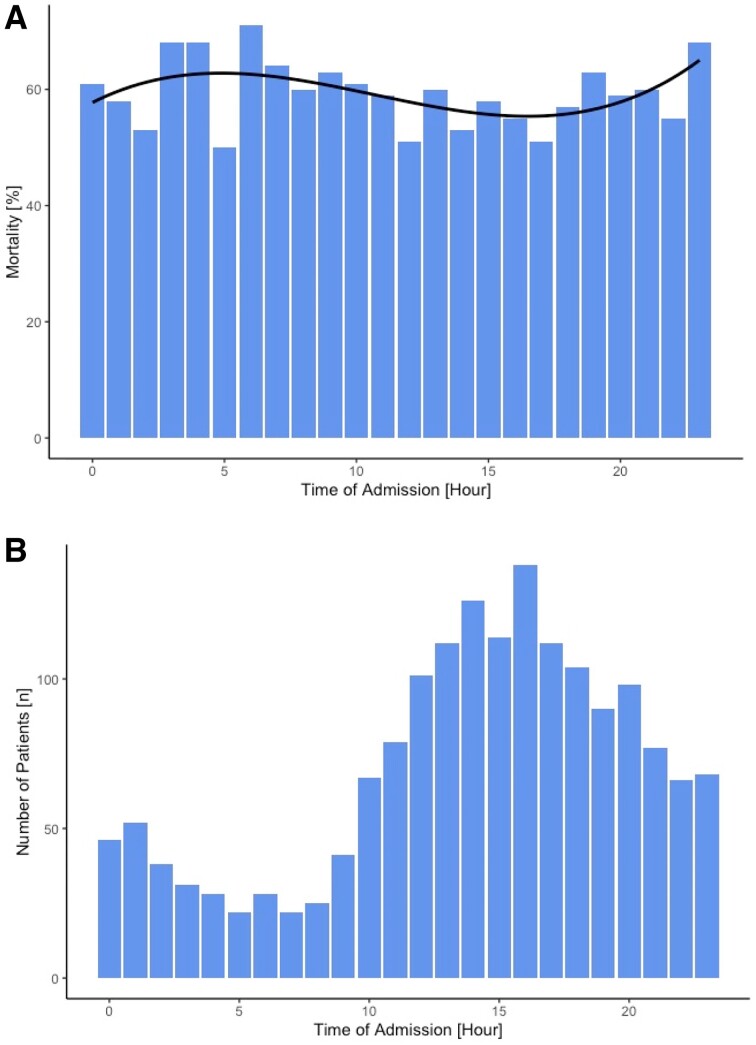
(*A*) Mortality (%) of all patients admitted at the respective time of day for each hour. (*B*) Number of patients (*n*) admitted at each hour over the course of a day.

In a second analysis, we calculated the 30-day mortality of patients stratified by the hour of admission as well as the number of admissions per hour (*Figure 2A* and *B*). The approximation curve of the mortality shows a bimodal distribution with the highest percentage of deaths occurring when admitted at 5 a.m. and 11 p.m. and a decline during on-hours, the lowest being in the afternoon. Using the Mann–Kendall test we were able to show a clear trend in the mortality over the course of a day with a Tau correlation coefficient of 1 and a *P*-value < 0.001.

**Figure 3:**
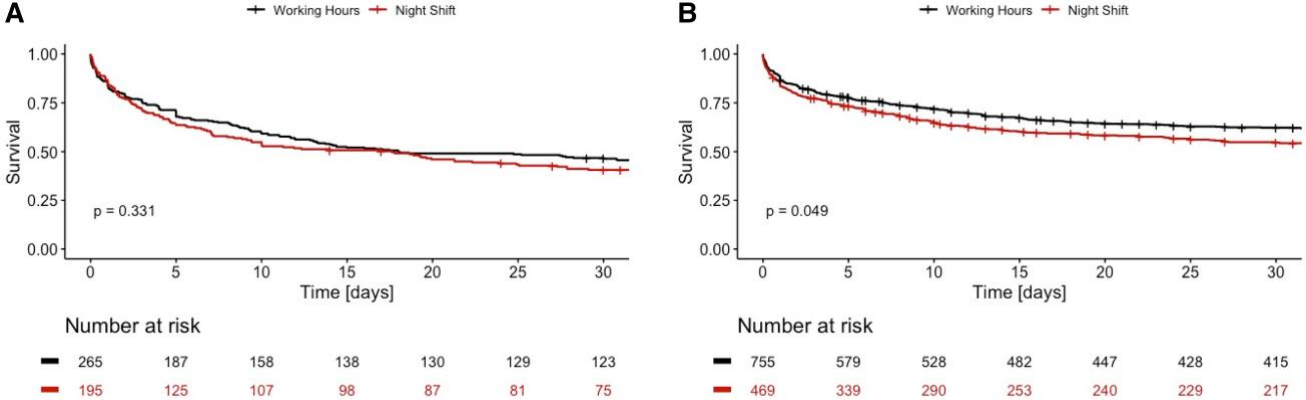
Cumulative survival curves for patients with VA-ECMO therapy (*A*) and without VA-ECMO (*B*) admitted during on-hours (black) or off-hours (red).

As depicted in *Figure 3A*, 30-day mortality rate did not differ in patients with a need for VA-ECMO therapy between an admission at on-hours and off-hours [HR 1.12 (0.89–1.40), *P* = 0.331]. Furthermore, there was no significant difference in mortality in patients requiring CPR between on- and off-hours [HR 1.14 (0.97–1.34), *P* = 0.114]. However, when looking at patients who did not require VA-ECMO treatment, a statistically significant increased 30-day mortality could be observed in patients admitted during off-hours as depicted in *Figure 3B* [HR 1.17 (1.00–1.36), *P* = 0.049]. Characteristics for patients stratified by VA-ECMO use are displayed in Supplementary material online, *Table S1*.

Patients with an acute myocardial infarction causing the cardiogenic shock did not have increased mortality during off-hours (*P* = 0.354). In contrast, those without an acute ischaemic aetiology presented with an increased mortality when admitted at off-hours [HR 1.27 (1.02–1.56), *P* = 0.029]. Characteristics for patients stratified by acute ischaemic aetiology are displayed in Supplementary material online, *Table S2*.

In accordance with the prior analyses, we could show that patients with a cardiogenic shock SCAI stage D or E resembling a severe form refractory to initial pharmacological therapy did not have an increased 30-day mortality when admitted during off-hours [HR 1.14 (0.98–1.41), *P* = 0.088]. The same accounts for patients with a classic cardiogenic shock SCAI stage C [HR 1.09 (0.82–1.45), *P* = 0.548]. Characteristics for patients stratified by SCAI stage are displayed in Supplementary material online, *Table S3*. Patients with lactate above the median lactate (4.4 mmol/L) in our cohort correlating with a more severe form of cardiogenic shock also showed no difference in mortality between an admission during on- and off-hours (*P* = 0.831). In contrast, in a pooled analysis patients with an initially less severe form of cardiogenic shock resembling an SCAI stage A or B exhibited a significantly increased mortality if presenting during off-hours [HR 2.23 (1.08–4.57), *P* = 0.029].

We performed a multivariable regression analysis with age, sex, and the need for CPR as potential confounders for the increased 30-day mortality in patients admitted at off-hours. Within this model, age (*P* < 0.001), CPR (*P* < 0.001) and an admission at off-hours (*P* = 0.047) presented as statistically significant predictors for an increased 30-day mortality rate while sex (*P* = 0.389) did not.

In a final step propensity score matching was performed using a logistic regression model with pH, lactate, and CPR as well as age and gender as matching parameters. In total 1345 patients had no missing values and were therefore eligible for propensity score matching. After propensity score matching groups were well balanced with a standard difference of mean below 0.15 for all matching parameters we were able to show that an admission during off-hours resulted in a statistically significant increased mortality compared to an admission during on-hours (*P* = 0.023).

## Discussion

In this study, we have shown that patients admitted with cardiogenic shock during off-hours had a increased 30-day mortality when compared to those presenting during on-hours.

Over the last years, there were only two studies evaluating the mortality of subjects depending on the time of admission in heart failure and cardiogenic shock. Cerbin et al. did not find a significant difference in mortality between admission at regular hours and off-hours in acute heart failure in their analysis.^11^ This study is difficult to compare with our work as only 4% of patients required the use of inotropes and almost none were treated with vasopressors. Consequently, most of the patients did not require intensive medical care treatment which is different from our cohort that consists of ICU patients only. Furthermore, as these were patients included in a randomized control trial assessing a new medication, they might have been given special attention during their stay in the hospital. This possible selection and performance bias also accounts for the results of the study by Sag et al. analyzing a subgroup of patients included in the CULPRIT-SHOCK trial concerning their individual mortality depending on the time of admission.^10^ The authors could not find a significant difference between an admission during on-hours and off-hours in cardiogenic shock patients. First of all, apart from the possible bias mentioned before, one has to recognize that only patients with an acute myocardial infarction and a multi-vessel coronary disease were included in this study. Within this specific subgroup of cardiogenic shock, we also did not observe any difference in mortality between an admission during on and off-hours. Secondly, 30% of those included in the study required mechanical circulatory support, 83% were intubated and mechanically ventilated, and almost all required vasopressors resembling an advanced SCAI stage. When comparing those seriously ill patients with equally severe forms of cardiogenic shock (SCAI stage D/E) in our cohort, we were not able to show a significant difference in 30-day mortality as well.

Recently a single-centre cohort study was published by Behnes et al. including 273 patients with cardiogenic shock showing an improved 30-day mortality in those admitted during off-hours. Interestingly, the authors could show an increased mortality in patients with acute myocardial infarction complicated by cardiogenic shock which is a result that we could not replicate in our cohort.^18^

Naturally, the treatment of cardiogenic shock has changed over the past two decades with the development of more advanced treatment options. Possibilities of haemodynamic monitoring including non-invasive and invasive strategies have changed over the last years e.g. with a broader use of pulmonary artery catheters. In terms of medical therapy various inotropic agents and vasopressors have been discussed controversially as different strategies have been proposed for the varying clinical presentations of cardiogenic shock.^19^ Probably the most dominant change in the therapy of cardiogenic shock is the widespread adoption of VA-ECMO for haemodynamic support even though a hereby decreased mortality is questionable.^20,21^ The increased use of VA-ECMO in cardiogenic shock can also be observed in our cohort with a total of 22 VA-ECMO being implanted between 2009 and 2012 which resembles 10–15% of patients with cardiogenic shock per year increasing to 30–45% in the following years.

Apart from the sub-study of the CULPRIT-SHOCK-Trial and the paper by Behnes et al. there are several studies looking into the question of a increased mortality in patients with acute coronary syndrome presenting during off-hours. A large meta-analysis by Sorita et al. proposed increased mortality during off-hours and an increased door-to-balloon time.^4^ These longer door-to-balloon times were also described in two other large studies.^22,23^ In contrast to that, two large registries could not find a difference in mortality between patients with acute coronary syndrome admitted during on-hours and off-hours suggesting the importance of STEMI networks.^6,24^ On a different note, Tokarek et al. suggested higher radiation doses as well as more periprocedural complications during percutaneous coronary interventions in patients presenting with a STEMI during off-hours.^25^ This increased rate of complications was replicated by a second study even though Sorita et al. found no difference in mortality.^5^ In line with the two registries, we were able to show that those with a cardiogenic shock as a result of an acute myocardial infarction did not have an increased 30-day mortality when being admitted to an ICU ward during off-hours.

Concerning CPR, Ofama et al. showed an increased mortality in patients being resuscitated during nights and at weekends compared to on-hours.^12^ Interestingly, looking at our data we could only find a numeric trend but no statistically significant difference in 30-day mortality between these two groups. Furthermore, the need for mechanical circulatory support (e.g. VA-ECMO) during off-hours also did not result in an increased mortality. Though at first glance irritating, we hypothesized that the implantation of an ECMO requires an interprofessional team with multiple personnel including senior physicians increasing the level of care for the individual patient. This is also in accordance with the current state of scientific knowledge as presented above.^13^

The same might account for patients with a severe form of cardiogenic shock corresponding to a SCAI stage D and E as those patients usually call for an intense care both from the medical as well as the nursing staff. In contrast, we could show that patients without ECMO therapy and with an SCAI stage A have an increased mortality when being admitted during off-hours. This might be caused by a prolonged time to diagnosis and time to treatment as also reported for other acute diseases like stroke and subarachnoid haemorrhage.^26,27^ The underlying reason could be that at first sight these patients might not appear as severely ill as they truly are. Furthermore, they might not require immediate medical attention resulting in a less optimal treatment at nighttime when resources both in terms of staffing and technologies are limited. Another reason might be that severely ill patients are being watched rather sensitively with an increased frequency of laboratory values as well as an increased time of medical attention and therefore it is more likely that a deteriorating course of disease is diagnosed and treated at an early timepoint. All of these possible explanations call for an improved risk stratification of patients with cardiogenic shock as currently used risk scores and classifications are being questioned for their predictive power and generalizability.^28^ Furthermore, this stresses the importance of a thorough workup of each patient admitted to the ICU ward and the clinical relevance of underestimating the severity of patients with only an initial stage of cardiogenic shock during a time of limited resources.

## Limitations

Finally, there are certain limitations concerning this study that need to be addressed. First, limitations that go along the retrospective character of this study such as a possible omitted variable bias, the inability to clearly define causality as well as the possibility of a data bias need to be mentioned. As patients between 2009 and 2022 were included it is important to mention that treatment of cardiogenic shock and its aetiologies has changed over time, which might have influenced our results. Furthermore, the reader is advised to take into account that patients admitted during off-hours had significantly lower pH and lactate levels and a higher percentage required CPR compared to those admitted during on-hours which might influence the 30-day mortality. Also, weekends and holidays were not assessed separately in the distinction between off-hours and on-hours. On top, regression analyses, especially when stratified by SCAI stages, might be difficult to generalize due to small sample sizes. However, to our knowledge, this study encompasses the largest cohort of cardiogenic shock patients treated on ICU wards to date.

## Conclusion

In conclusion, we were able to show a increased 30-day mortality in patients with cardiogenic shock admitted during off-hours. This seems to be mainly driven by patients with a milder onset of disease calling on the one hand for a thorough workup of each patient with cardiogenic shock admitted to the ICU ward. On the other hand, it shows the urgent need for improved risk stratification measures and scores. Therefore, further studies assessing tools to predict the mortality as well as the severe course of disease of patients with cardiogenic shock are warranted based on the results of this study.

## Supplementary Material

zuae012_Supplementary_Data

## Data Availability

The data underlying this article will be shared on reasonable request to the corresponding author.

## References

[1] Chioncel O , Parissis J, Mebazaa A, Thiele H, Desch S, Bauersachs J, et al Epidemiology, pathophysiology and contemporary management of cardiogenic shock—a position statement from the Heart Failure Association of the European Society of Cardiology. Eur J Heart Fail 2020;22:1315–1341. 32469155 10.1002/ejhf.1922

[2] Rathod KS , Koganti S, Iqbal MB, Jain AK, Kalra SS, Astroulakis Z, et al Contemporary trends in cardiogenic shock: incidence, intra-aortic balloon pump utilisation and outcomes from the London heart attack group. Eur Heart J Acute Cardiovasc Care 2018;7:16–27. 29111770 10.1177/2048872617741735

[3] Yu YY , Zhao BW, Ma L, Dai XC. Association between out-of-hour admission and short- and long-term mortality in acute myocardial infarction: a systematic review and meta-analysis. Front Cardiovasc Med 2021;8:752675. 34970604 10.3389/fcvm.2021.752675 PMC8712470

[4] Sorita A , Ahmed A, Starr SR, Thompson KM, Reed DA, Prokop L, et al Off-hour presentation and outcomes in patients with acute myocardial infarction: systematic review and meta-analysis. BMJ 2014;348:f7393. 24452368 10.1136/bmj.f7393 PMC3898160

[5] Sorita A , Lennon RJ, Haydour Q, Ahmed A, Bell MR, Rihal CS, et al Off-hour admission and outcomes for patients with acute myocardial infarction undergoing percutaneous coronary interventions. Am Heart J 2015;169:62–68. 25497249 10.1016/j.ahj.2014.08.012

[6] Casella G , Ottani F, Ortolani P, Guastaroba P, Santarelli A, Balducelli M, et al Off-hour primary percutaneous coronary angioplasty does not affect outcome of patients with ST-segment elevation acute myocardial infarction treated within a regional network for reperfusion: the REAL (registro regionale angioplastiche dell’Emilia-romagna) registry. JACC Cardiovasc Interv 2011;4:270–278. 21435603 10.1016/j.jcin.2010.11.012

[7] Mrochen A , Sprügel MI, Gerner ST, Madžar D, Kuramatsu JB, Hoelter P, et al Invasiveness and clinical outcomes of off-hour admissions in patients with intracerebral hemorrhage. J Stroke Cerebrovasc Dis Off J Natl Stroke Assoc 2020;29:104505. 10.1016/j.jstrokecerebrovasdis.2019.104505 31786043

[8] You JS , Park YS, Chung SP, Lee HS, Jeon S, Kim WY, et al Relationship between time of emergency department admission and adherence to the surviving sepsis campaign bundle in patients with septic shock. Crit Care Lond Engl 2022;26:43. 10.1186/s13054-022-03899-0 PMC8832860 35148797

[9] Aoki M , Abe T, Matsumura Y, Hagiwara S, Saitoh D, Oshima K. The off-hour effect among severe trauma patients: a nationwide cohort study in Japan. Surg Today 2020;50:1480–1485. 32458232 10.1007/s00595-020-02027-1

[10] Sag CM , Zeymer U, Ouarrak T, Schneider S, Montalescot G, Huber K, et al Effects of ON-hours versus OFF-hours admission on outcome in patients with myocardial infarction and cardiogenic shock: results from the CULPRIT-SHOCK trial. Circ Cardiovasc Interv 2020;13:e009562. 32883104 10.1161/CIRCINTERVENTIONS.120.009562

[11] Cerbin LP , Ambrosy AP, Greene SJ, Armstrong PW, Butler J, Coles A, et al Is time of the essence? The impact of time of hospital presentation in acute heart failure: insights from ASCEND-HF trial. JACC Heart Fail 2018;6:298–307. 29525328 10.1016/j.jchf.2018.01.018 PMC7445462

[12] Ofoma UR , Basnet S, Berger A, Kirchner HL, Girotra S. American heart association get with the guidelines—resuscitation investigators. Trends in survival after in-hospital cardiac arrest during nights and weekends. J Am Coll Cardiol 2018;71:402–411. 29389356 10.1016/j.jacc.2017.11.043 PMC5858924

[13] Gómez-Sánchez R , García-Carreño J, Martínez-Solano J, Sousa-Casasnovas I, Juárez-Fernández M, Devesa-Cordero C, et al Off-Hours versus regular-hours implantation of peripheral venoarterial extracorporeal membrane oxygenation in patients with cardiogenic shock. J Clin Med 2023;12:1875. 36902662 10.3390/jcm12051875 PMC10003377

[14] Schrage B , Dabboura S, Yan I, Hilal R, Neumann JT, Sörensen NA, et al Application of the SCAI classification in a cohort of patients with cardiogenic shock. Catheter Cardiovasc Interv Off J Soc Card Angiogr Interv 2020;96:E213–E219. 10.1002/ccd.28707 31925996

[15] Baran DA , Grines CL, Bailey S, Burkhoff D, Hall SA, Henry TD, et al SCAI clinical expert consensus statement on the classification of cardiogenic shock: this document was endorsed by the American College of Cardiology (ACC), the American Heart Association (AHA), the Society of Critical Care Medicine (SCCM), and the Society of Thoracic Surgeons (STS) in April 2019. Catheter Cardiovasc Interv Off J Soc Card Angiogr Interv 2019;94:29–37. 10.1002/ccd.28329 31104355

[16] Hansen BB . Full matching in an observational study of coaching for the SAT. J Am Stat Assoc 2004;99:609–618.

[17] Stuart EA , Green KM. Using full matching to estimate causal effects in nonexperimental studies: examining the relationship between adolescent marijuana use and adult outcomes. Dev Psychol 2008;44:395–406. 18331131 10.1037/0012-1649.44.2.395 PMC5784842

[18] Behnes M , Rusnak J, Egner-Walter S, Ruka M, Dudda J, Schmitt A, et al Effect of admission and onset time on the prognosis of patients with cardiogenic shock. Chest 2023;165:110–127. S0012-3692(23)05270-4. 37579943 10.1016/j.chest.2023.08.011

[19] Bruno RR , Wolff G, Kelm M, Jung C. Pharmacological treatment of cardiogenic shock—a state of the art review. Pharmacol Ther 2022;240:108230. 35697151 10.1016/j.pharmthera.2022.108230

[20] Thiele H , Zeymer U, Akin I, Behnes M, Rassaf T, Mahabadi AA, et al Extracorporeal life support in infarct-related cardiogenic shock. N Engl J Med 2023;389:1286–1297. 37634145 10.1056/NEJMoa2307227

[21] Lim HS , Howell N, Ranasinghe A. Extracorporeal life support: physiological concepts and clinical outcomes. J Card Fail 2017;23:181–196. 27989868 10.1016/j.cardfail.2016.10.012

[22] Shavelle DM , Zheng L, Ottochian M, Wagman B, Testa N, Hall S, et al Time of day variation in door-to-balloon time for STEMI patients in Los Angeles county: does time of day make a difference? Acute Card Care 2013;15:52–57. 23738606 10.3109/17482941.2013.776690

[23] Cubeddu RJ , Palacios IF, Blankenship JC, Horvath SA, Xu K, Kovacic JC, et al Outcome of patients with ST-segment elevation myocardial infarction undergoing primary percutaneous coronary intervention during on- versus off-hours (a harmonizing outcomes with revascularization and stents in acute myocardial infarction [HORIZONS-AMI] trial substudy). Am J Cardiol 2013;111:946–954. 23340031 10.1016/j.amjcard.2012.11.062

[24] Tscharre M , Jäger B, Farhan S, Christ G, Schreiber W, Weidinger F, et al Impact of time of admission on short- and long-term mortality in the Vienna STEMI registry. Int J Cardiol 2017;244:1–6. 28784440 10.1016/j.ijcard.2017.03.029

[25] Tokarek T , Dziewierz A, Plens K, Rakowski T, Jaroszyńska A, Bartuś S, et al Percutaneous coronary intervention during on- and off-hours in patients with ST-segment elevation myocardial infarction. Hell J Cardiol HJC Hell Kardiologike Epitheorese 2021;62:212–218. 10.1016/j.hjc.2021.01.011 33540055

[26] Zha M , Yang Q, Liu S, Wu M, Huang K, Cai H, et al Off-hour effect on time metrics and clinical outcomes in endovascular treatment for large vessel occlusion: a systematic review and meta-analysis. Int J Stroke Off J Int Stroke Soc 2022;17:669–680. 10.1177/17474930211012545 33877016

[27] Jun SM , Kim SH, Leinonen H, Gan P, Bhat S. Impact of off-hour admission with subarachnoid hemorrhage: a meta-analysis. World Neurosurg 2022;166:e872–e891. 35948214 10.1016/j.wneu.2022.07.127

[28] Beer BN , Jentzer JC, Weimann J, Dabboura S, Yan I, Sundermeyer J, et al Early risk stratification in patients with cardiogenic shock irrespective of the underlying cause—the cardiogenic shock score. Eur J Heart Fail 2022;24:657–667. 35119176 10.1002/ejhf.2449

