# Supplementary material for: The association of off-hour vs. on-hour intensive care unit admission time with mortality in patients with cardiogenic shock: a retrospective multi-centre analysis
Source: Eur Heart J Acute Cardiovasc Care. 2024 Feb 2;13(4):347–53. doi: 10.1093/ehjacc/zuae012 (PMC11075929; doi:10.1093/ehjacc/zuae012)
Supplement: zuae012_Supplementary_Data [file zuae012_supplementary_data.docx]

|  | VA-ECMO (n = 460) | No VA-ECMO (n = 1225) | p-Value |
| --- | --- | --- | --- |
| Age (years) | 58 (50 – 66) | 73 (60 – 80) | <0.001* |
| Sex |  |  |  |
| Male (n) | 372 (81 %) | 871 (71 %) | <0.001* |
| Female (n) | 88 (19 %) | 353 (29 %) |  |
| Cardiopulmonary Rescucitation (n) | 314 (68 %) | 597 (49 %) | <0.001* |
| Vasopressors & Inotropes (n) | 452 (98 %) | 1092 (89 %) | <0.001* |
| Dialysis (n) | 184 (40 %) | 299 (24 %) | <0.001* |
| Laboratory Values |  |  |  |
| pH | 7.3 (7.2 – 7.4) | 7.3 (7.2 – 7.4) | <0.001* |
| Lactate (mmol/L) | 9.5 (3.7 – 14.8) | 3.4 (2.0 – 7.8) | <0.001* |
| Hemoglobin (mg/dL) | 10.2 (9.2 – 11.7) | 11.2 (9.6 – 13.0) | <0.001* |
| Creatinine (mg/dL) | 1.6 (1.1 – 2.0) | 1.5 (1.1 – 2.1) | 0.51 |
| Vital Parameters |  |  |  |
| Heart Rate (bpm) | 104 (86 – 120) | 94 (80 – 112) | <0.001* |
| Systolic Blood Pressure (mmHg) | 80 (67 – 90) | 90 (75 – 100) | <0.001* |
| Diastolic Blood Pressure (mmHg) | 50 (40 – 60) | 48 (40 – 55) | 0.002* |
| Intracranial Bleeding (n) | 54 (12 %) | 48 (4 %) | <0.001* |
| Ischemic Stroke (n) | 49 (11 %) | 101 (8 %) | 0.23 |
| Hemorrhagic Stroke (n) | 7 (2 %) | 9 (1 %) | 0.09 |
| Bleeding BARC ≥2 (n) | 296 (64 %) | 374 (31 %) | <0.001* |
| Sepsis (n) | 96 (21 %) | 156 (13 %) | <0.001* |

**Supplementary Table 1 – Subgroup of patients with and without VA-ECMO**

All values presented as median and confidence intervals or absolute values and percentages. VA-ECMO = venoarterial extracorporeal membrane oxygenation, pH = potential of hydrogen, BARC = Bleeding Academic Research Consortium. * = p-values <0.05 were considered statistically significant

|  | Acute myocardial infarction (n = 837) | No acute myocardial infarction (n = 784) | p-Value |
| --- | --- | --- | --- |
| Age (years) | 69 (58 – 78) | 68 (54 – 78) | 0.04* |
| Sex |  |  | 0.008* |
| Male (n) | 643 (77 %) | 556 (71 %) |  |
| Female (n) | 194 (23 %) | 228 (29 %) |  |
| SCAI |  |  | 0.02* |
| A (n) | 7 (0.8 %) | 4 (0.5 %) |  |
| B (n) | 29 (3.5 %) | 33 (4.2 %) |  |
| C (n) | 227 (27 %) | 262 (33 %) |  |
| D (n) | 130 (16 %) | 133 (17 %) |  |
| E (n) | 432 (52 %) | 3 (0.4 %) |  |
| Cardiopulmonary Rescucitation (n) | 547 (65 %) | 336 (43 %) | <0.001* |
| Vasopressors & Inotropes (n) | 785 (94 %) | 706 (90 %) | 0.01* |
| VA-ECMO (n) | 264 (32 %) | 178 (23 %) | <0.001* |
| Dialysis (n) | 232 (28 %) | 220 (28 %) | 0.56 |
| Laboratory Values |  |  |  |
| pH | 7.3 (7.2 – 7.3) | 7.3 (7.2 – 7.4) | <0.001* |
| Lactate (mmol/L) | 6.6 (2.8 – 11.8) | 3.1 (1.9 – 7.2) | <0.001* |
| Hemoglobin (mg/dL) | 10.7 (9.4 – 12.9) | 11 (9.5 – 12.5) | 0.82 |
| Creatinine (mg/dL) | 1.4 (1.1 – 1.9) | 1.6 (1.1 – 2.3) | 0.004* |
| Vital Parameters |  |  |  |
| Heart Rate (bpm) | 100 (84 – 116) | 95 (80 – 112) | 0.003* |
| Systolic Blood Pressure (mmHg) | 85 (70 – 98) | 90 (75 – 100) | <0.001* |
| Diastolic Blood Pressure (mmHg) | 48 (40 – 55) | 50 (40 – 55) | 0.77 |
| Intracranial Bleeding (n) | 59 (7 %) | 41 (5%) | 0.19 |
| Ischemic Stroke (n) | 65 (8%) | 82 (11 %) | 0.17 |
| Hemorrhagic Stroke (n) | 7 (1 %) | 7 (1 %) | 0.58 |
| Bleeding BARC ≥2 (n) | 347 (42 %) | 310 (40 %) | 0.44 |
| Sepsis (n) | 111 (14 %) | 125 (16 %) | 0.25 |

**Supplementary Table 2 – Subgroup of patients with and without an acute coronary syndrome**

All values presented as median and confidence intervals or absolute values and percentages. SCAI Classification = Society for Cardiovascular Angiography & Interventions Stages of Cardiogenic Shock, VA-ECMO = venoarterial extracorporeal membrane oxygenation, pH = potential of hydrogen, BARC = Bleeding Academic Research Consortium. * = p-values <0.05 were considered statistically significant

|  | SCAI A (n = 11) | SCAI B (n = 62) | SCAI C (n = 538) | SCAI D (n = 265) | SCAI E (n = 786) | p-Value |
| --- | --- | --- | --- | --- | --- | --- |
| Age (years) | 70 (63 – 85) | 74 (63 – 82) | 70 (57 – 79) | 70 (56 – 80) | 65 (54 – 78) | <0.001* |
| Sex |  |  |  |  |  | 0.88 |
| Male (n) | 7 (64 %) | 44 (71 %) | 397 (74 %) | 185 (70 %) | 592 (75 %) |  |
| Female (n) | 4 (36 %) | 18 (29 %) | 141 (26 %) | 80 (30 %) | 193 (25 %) |  |
| Cardiopulmonary Rescucitation (n) | 1 (9 %) | 13 (21 %) | 203 (38 %) | 133 (50 %) | 561 (71 %) | <0.001* |
| Vasopressors & Inotropes (n) | 6 (55 %) | 44 (71 %) | 480 (89 %) | 254 (96 %) | 758 (96 %) | < 0.001* |
| VA-ECMO (n) | 0 (0 %) | 1 (2 %) | 42 (8 %) | 51 (19 %) | 362 (46 %) | <0.001* |
| Dialysis (n) | 3 (27 %) | 8 (13 %) | 131 (24 %) | 70 (26 %) | 268 (34 %) | 0.001 |
| Laboratory Values |  |  |  |  |  |  |
| pH | 7.4 (7.3 – 7.4) | 7.4 (7.3 – 7.4) | 7.3 (7.3 – 7.4) | 7.3 (7.2 – 7.4) | 7.3 (7.2 – 7.3) | <0.001* |
| Lactate (mmol/L) | 1.7 (1.1 – 1.9) | 1.5 (1.0 – 1.3) | 2.3 (1.6 – 3.8) | 3.1 (2.0 – 7.4) | 8.1 (4.1 – 13.4) | <0.001* |
| Hemoglobin (mg/dL) | 9.8 (9.2 – 11.5) | 11.3 (9.7 – 12.8) | 11.4 (9.9 – 13.1) | 10.9 (9.5 – 12.6) | 10.5 (9.3 – 12.3) | <0.001* |
| Creatinine (mg/dL) | 1.1 (0.8 – 1.8) | 1.2 (1.0 – 2.0) | 1.3 (1.0 – 1.8) | 1.7 (1.2 – 2.5) | 1.6 (1.2 – 2.1) | <0.001* |
| Vital Parameters |  |  |  |  |  |  |
| Heart Rate (bpm) | 88 (76 – 102) | 90 (76 – 106) | 92 (78 – 110) | 96 (80 – 112) | 100 (84 – 120) | <0.001* |
| Systolic Blood Pressure (mmHg) | 105 (94 – 111) | 95 (81 – 110) | 90 (80 – 104) | 89 (75 – 98) | 80 (70 – 95) | <0.001* |
| Diastolic Blood Pressure (mmHg) | 55 (41 – 59) | 49 (40 – 55) | 50 (40 – 59) | 45 (40 – 55) | 48 (39 – 55) | <0.001* |
| Intracranial Bleeding (n) | 1 (9 %) | 3 (5 %) | 16 (3 %) | 18 (7 %) | 64 (8 %) | 0.06 |
| Ischemic Stroke (n) | 2 (18 %) | 6 (10 %) | 40 (7 %) | 29 (11 %) | 71 (9 %) | 0.58 |
| Hemorrhagic Stroke (n) | 0 (0 %) | 0 (0 %) | 5 (1 %) | 4 (2 %) | 7 (1 %) | 0.96 |
| Bleeding BARC ≥2 (n) | 5 (46 %) | 15 (24 %) | 141 (26 %) | 104 (39 %) | 401 (51 %) | <0.001* |
| Sepsis (n) | 3 (27 %) | 4 (7 %) | 67 (13 %) | 36 (14 %) | 140 (18 %) | 0.004* |

**Supplementary Table 3 – Subgroups according to SCAI stage**

All values presented as median and confidence intervals or absolute values and percentages. SCAI Classification = Society for Cardiovascular Angiography & Interventions Stages of Cardiogenic Shock, VA-ECMO = venoarterial extracorporeal membrane oxygenation, pH = potential of hydrogen, BARC = Bleeding Academic Research Consortium. * = p-values <0.05 were considered statistically significant


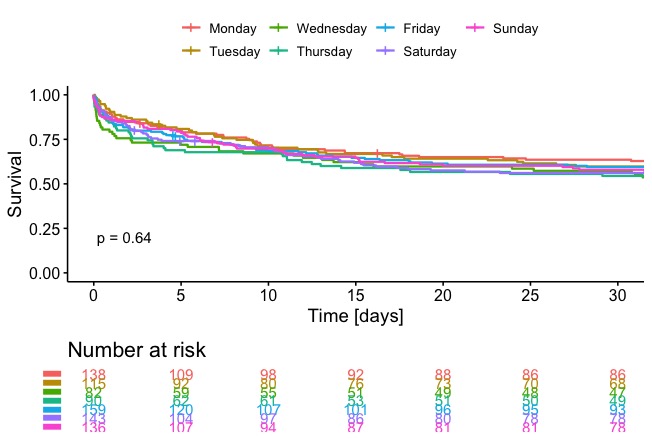

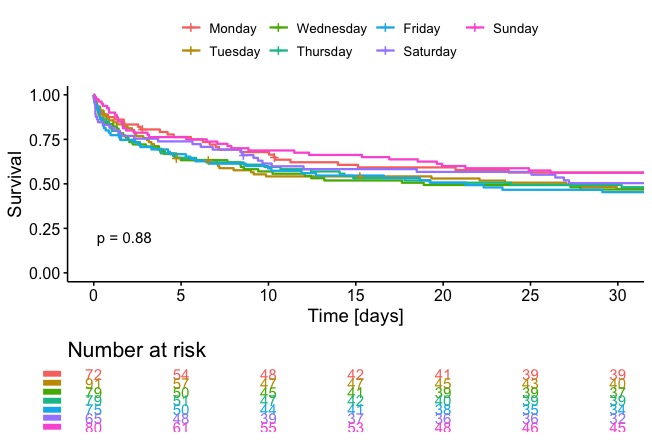


A)

B)

**Supplementary Figure 1 – Mortality in dependance of the day of the week of the patient´s admission**

Cumulative survival curves for patients admitted during working hours (A) and off-hours (B) for each day of the week.
